# Inhibition Mechanism of Chitooligosaccharide-Polyphenol Conjugates toward Polyphenoloxidase from Shrimp Cephalothorax

**DOI:** 10.3390/molecules28145560

**Published:** 2023-07-20

**Authors:** Ajay Mittal, Avtar Singh, Bin Zhang, Qiancheng Zhao, Soottawat Benjakul

**Affiliations:** 1 International Center of Excellence in Seafood Science and Innovation, Faculty of Agro-Industry, Prince of Songkla University, Hat Yai 90110, Thailand; ajy.mittal@yahoo.com (A.M.); avtar.s@psu.ac.th (A.S.); 2 Key Laboratory of Health Risk Factors for Seafood of Zhejiang Province, College of Food Science and Pharmacy, Zhejiang Ocean University, Zhoushan 316022, China; zhangbin@zjou.edu.cn; 3 School of Food Science and Engineering, Dalian Ocean University, Dalian 116023, China; qczhao@dlou.edu.cn; 4 Department of Food and Nutrition, Kyung Hee University, Seoul 02447, Republic of Korea

**Keywords:** chitooligosaccharide-catechin, conjugate, shrimp cephalothorax, polyphenoloxidase, enzyme inhibition, binding interactions

## Abstract

Crustaceans are perishable with a short shelf-life. They are prone to deterioration after capture, particularly during handling, processing, and storage due to melanosis caused by polyphenoloxidase (PPO). Therefore, inhibitory effects of chitooligosaccharide (CHOS) in comparison with CHOS-catechin (CHOS-CAT), CHOS-epigallocatechin gallate (CHOS-EGCG), and CHOS-gallic acid (CHOS-GAL) conjugates on Pacific white shrimp cephalothorax PPO were studied. IC_50_ of CHOS-CAT (0.32 mg/mL) toward PPO was less than those of all conjugates tested (*p* < 0.05). CHOS-CAT exhibited the mixed-type inhibition. *K_ic_* (0.58 mg/mL) and *K_iu_* (0.02 mg/mL) of CHOS-CAT were lower than those of other conjugates (*p* < 0.05). CHOS-CAT showed static fluorescence-quenching, suggesting a change in micro-environment around the active site of PPO. Moreover, CHOS-CAT was linked with various amino acid residues, including Tyr208 or Tyr209 of proPPO via van der Waals, hydrophobic interaction, and hydrogen bonding as elucidated by the molecular docking of proPPO. Although CHOS-CAT had the highest PPO inhibitory activity, it showed a lower binding energy (−8.5 kcal/mol) than other samples, except for CHOS-EGCG (−10.2 kcal/mol). Therefore, CHOS-CAT could act as an anti-melanosis agent in shrimp and other crustaceans to prevent undesirable discoloration associated with quality losses.

## 1. Introduction

Pacific white shrimp (*Litopenaeus vannamei*) account for over 90% of worldwide farmed shrimp production and has gained increasing economic importance due to its savory taste. However, shrimps are considered a highly perishable seafood commodity due to their vulnerability to discoloration (melanosis) and microbial spoilage. Those phenomena generally limit shelf-life and lower consumer acceptability. Moreover, undesirable melanosis majorly causes enormous economic loss to the shrimp processing industry. Thus, the control of melanosis is crucial for shrimp processors or farmers to maintain the quality and acceptability related to market value.

Polyphenoloxidase (PPO) or tyrosinase, a type-3 copper protein, consists of two Cu atoms at the active site, and each Cu atom is flanked by three histidine residues [1,2]. PPO or tyrosinase in crustaceans cause melanosis by inducing hydroxylation of phenols (tyrosine in shrimp) to 3-(3,4-dihydroxyphenyl)propionic acid (DOPA), which is then converted to DOPA-quinones in the presence of oxygen [3]. Subsequently, non-enzymatic self-polymerization of DOPA-quinones causes the formation of leucodopachrome, which is rapidly oxidized to dopachrome. Thereafter, 5,6-dihydroxyindole formed from dopachrome is further oxidized to indole-5,6-quinones and then polymerized to form melanin [4]. Several compounds have been employed as PPO inhibitors, including chelating agent (ethylene diamine tetra acetic acid), ascorbic acid, hydrogen sulfite, sodium metabisulfite, and 4-hexylresorcinol [5]. Plant extracts rich in polyphenolic compounds have been employed as safe natural PPO inhibitors, e.g., dechlorophyllized green tea extract [6], dechlorophyllized Chamuang leaf extract [7], dechlorophyllized guava leaf extract [8], etc. Those extracts could retard the formation of melanosis in Pacific white shrimp during the refrigerated/chilled storage. Furthermore, fruiting body extract of mushroom was reported to inhibit PPO [9]. Among aforesaid PPO inhibitors, sodium metabisulfite is used mostly, nevertheless it is associated with health-related issues such as allergy or serious ailment in asthmatic patients [10]. Among PPO inhibitors, 4-hexylresorcinol has higher chemical stability, specificity, and efficacy at low doses without bleaching effect on treated crustaceans. Moreover, 4-hexylresorcinol is considered Generally Recognized As Safe (GRAS) and has been approved as an additive in shrimp by the Food and Drug Administration, USA [4]. However, 4-hexylresorcinol is still costly for large-scale processing [4,5]. Considering the health and safety of consumers, PPO inhibitors derived from nature have received extensive attention from shrimp processors or farmers. Plant polyphenols (PPNs), such as catechin, epicatechin gallate, ferulic acid, epicatechin, epigallocatechin gallate, and epigallocatechin, or phenolic extracts from plants have been used as alternative anti-melanosis agents or PPO inhibitors due to their non-toxic nature [4]. Moreover, chitosan (CS) and chitooligosaccharide (CHOS) also showed PPO inhibitory activity in different food matrices [11,12]. In addition, CHOS based derivatives, such as CHOS-PPN conjugates possessed more enhanced bioactivities (antioxidant and antimicrobial activities) than native CHOS [13]. Considering the efficacy of polyphenols and CHOS in the inhibition of PPO, CHOS-PPN conjugates could act as a potential novel inhibitor of PPO, which could prevent melanosis in shrimp or other crustaceans.

Therefore, the present investigation aimed to examine the inhibitory efficacy and inhibition mechanism of CHOS and various CHOS-PPN conjugates toward PPO from Pacific white shrimp. The kinetics inhibition as well as conformational changes of PPO induced by CHOS or CHOS-PPN conjugates were also determined. Molecular docking was performed to elucidate the various interactions between the active site of proPPO and CHOS or CHOS-PPN conjugates, and the binding free energies were also computed.

## 2. Results and Discussion

### 2.1. Inhibitory Effect of CHOS or Different CHOS-PPN Conjugates on Shrimp PPO 

All samples showed inhibition against PPO in a concentration-dependent manner (*p* < 0.05) (Figure S1A). Irrespective of the concentrations used, all conjugate samples showed higher PPO inhibitory activity than CHOS (*p* < 0.05). This indicated that PPN conjugation on CHOS backbone significantly enhanced PPO inhibitory activity. When the highest concentration (1 mg/mL) of the sample was used, CHOS-CAT showed the highest inhibitory activity towards PPO (58.06%), while CHOS-EGCG (53.20%), CHOS-GAL (50.95%), and CHOS (29.74%) had lower inhibition (*p* < 0.05). Also, phenolic compounds are able to inhibit PPO activity by interacting with the active sites of the enzymes or via reduction in quinone formed. Catechin and its various derivatives (epicatechin, epicatechin gallate, epigallocatechin, and EGCG) showed PPO inhibition between 15 and 60% when used at 2.0 mM [14]. Ferulic acid with different concentrations (0.1–2.0%, *w*/*v*) showed inhibitory activity towards Pacific white shrimp PPO in a dose dependent manner [15].

In addition, IC_50_ values against PPO of CHOS-CAT, CHOS-EGCG, and CHOS-GAL conjugates were 0.32, 0.39, and 0.96 mg/mL, respectively (Figure S1B). Among all samples, CHOS-CAT had the lowest IC_50_, whereas CHOS-GAL exhibited the highest value (*p* < 0.05). Moreover, IC_50_ of CHOS could not be determined since PPO inhibition could not reach 50% at all tested concentrations (Figure S1A). The result indicated the low efficacy in inhibiting PPO by CHOS. The lower IC_50_ of CHOS-CAT toward PPO was more likely due to its higher specificity and efficacy due to the conjugation of CAT on CHOS backbone. CAT possessed two benzene rings namely ring A and ring B, in which each ring has two hydroxyl groups. Additionally, a hydroxyl group is present at C3 of the C ring (dihydropyran heterocyclic ring). EGCG consists of eight hydroxyl groups and a galloyl moiety at the C3 position in the C ring [16]. However, the presence of gallate moiety was plausibly related to the bulky structure of EGCG, which might not fit properly in the pocket of PPO active site. As a result, it contributed to lower PPO inhibitory activity. Similarly, despite its small size, GAL has less hydroxyl groups than EGCG and CAT [16,17,18], which could contribute to lower interactions with amino acid residues present at the active site of PPO. 

**Figure 1:**
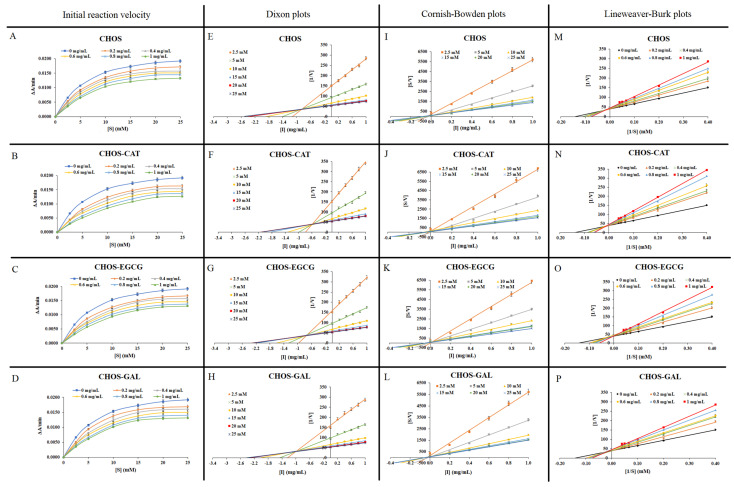
Initial reaction velocity in the presence of L-DOPA at different concentrations (**A**–**D**), Dixon plots (**E**–**H**), Cornish-Bowden plots (**I**–**L**), and Lineweaver-Burk plots (**M**–**P**) for PPO inhibition by CHOS and different CHOS-PPN conjugates at varying concentrations. Bars represent standard deviation (n = 3). CHOS: chitooligosaccharide; CHOS-CAT: chitooligosaccharide-catechin; CHOS-EGCG: chitooligosaccharide-epigallocatechin gallate; and CHOS-GAL: chitooligosaccharide-gallic acid.

The inhibitory impact of CHOS and its conjugates at different concentrations on PPO was elucidated by the determination of the initial reaction rate through the formation of dopachrome using substrate (L-DOPA) at various concentrations. The initial reaction rates were decreased with augmenting concentrations of CHOS, CHOS-CAT, CHOS-EGCG, and CHOS-GAL. Irrespective of L-DOPA concentrations, the inhibition of PPO activity was sample dose-dependent (Figure 1A–D). The type of PPO inhibition caused by CHOS, CHOS-CAT, CHOS-EGCG, and CHOS-GAL was deduced using Dixon (D) and CB plots (Figure 1E–H and Figure 1I–L, respectively). All samples in D or CB plots intersected in the second quadrant at a single point, regardless of L-DOPA concentration, indicating mixed-type kinetics (both competitive and un-competitive) for PPO inhibition. In general, the competitive inhibition constant (*K_ic_*) is determined in the second quadrant of the D plot, where extrapolated lines are intersected [13]. Similarly, the un-competitive inhibition constant (*K_iu_*) is computed in the second quadrant of the CB plot where extrapolated lines are intersected. *K_ic_* and *K_iu_* (0.58 and 0.02 mg/mL, respectively) of CHOS-CAT were less than those of other samples (*p* < 0.05). CHOS-EGCG, CHOS-GAL, and CHOS had *K_ic_* of 0.72, 0.83, and 0.94 mg/mL and *K_iu_* of 0.02, 0.02, and 0.04 mg/mL, respectively. Generally, the low *K_ic_* of an inhibitor is related to high enzyme inhibitory activity, while a high *K_ic_* reflects weak inhibitory activity. Nevertheless, *K_iu_* did not differ among all conjugate samples (*p* > 0.05), whereas CHOS had the highest *K_iu_* (*p* < 0.05). In addition, a lower value of *K_iu_* than *K_ic_* indicated that the inhibitor had a greater complexation with the enzyme-substrate complex than the free enzyme. This character is found in all samples. Thus, among all samples, CHOS-CAT showed both un-competitive inhibition and competitive inhibition, as witnessed by a lower *K_ic_* and *K_iu_* than other samples (Table 1). The result was also supported by the lower IC_50_ value for CHOS-CAT (Figure S1B). 

*K_m_* and *V_max_* values of CHOS and its conjugates calculated based on the Lineweaver-Burk (LB) plot (Figure 1M–P) are given in Table 1. The extrapolated linear lines were intersected in the second quadrant of the graph at a single point, in which *V_max_* and *K_m_* were decreased and increased, respectively, (*p* < 0.05) when the concentration of all CHOS-PPN conjugates was augmented. This result revealed both competitive and un-competitive inhibition known as mixed-type inhibition. Likewise, CHOS also exhibited mixed-type inhibition toward PPO activity. The result was more likely obtained from D and CB plots. LB plot is more often used to determine the inhibition type, whereas D and CB plots have been implemented to confirm the inhibition type.

**Figure 2:**
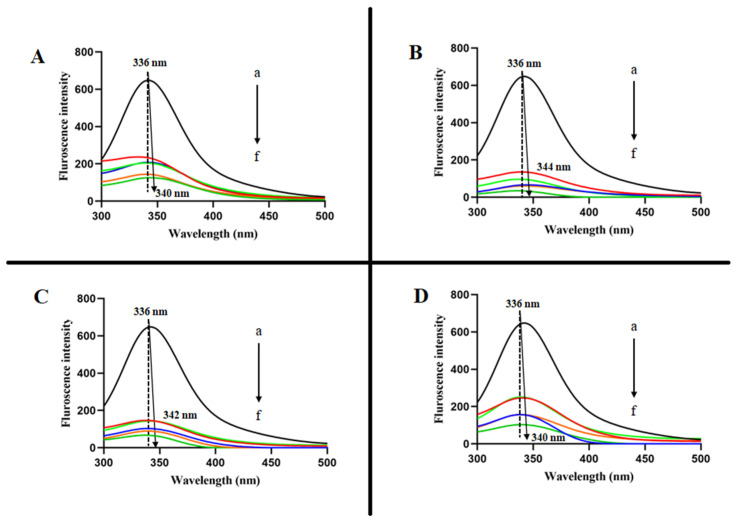
Fluorescence spectra of PPO in the presence of CHOS (**A**), CHOS-CAT (**B**), CHOS-EGCG (**C**), CHOS-GAL (**D**) at 37 °C. a→f shows an increase in concentration of A, B, C, and D from 0 to 1 mg/mL. CHOS: chitooligosaccharide; CHOS-CAT: chitooligosaccharide-catechin; CHOS-EGCG: chitooligosaccharide-epigallocatechin gallate; and CHOS-GAL: chitooligosaccharide-gallic acid.

The normalized fluorescence-emission spectra of PPO in the presence of CHOS and its conjugates at different concentrations are shown in Figure 2. The intensity of the emission band at 336 nm, corresponding to aromatic residues (Phe, Trp, and Tyr) in PPO [19,20] was decreased with the increasing concentration of all samples. The result showed that all samples could extinguish the intrinsic fluorescence of PPO. Moreover, at higher concentrations of samples, a red shift was observed, indicating a change in micro-surroundings around aromatic amino acids of PPO.

**Figure 3:**
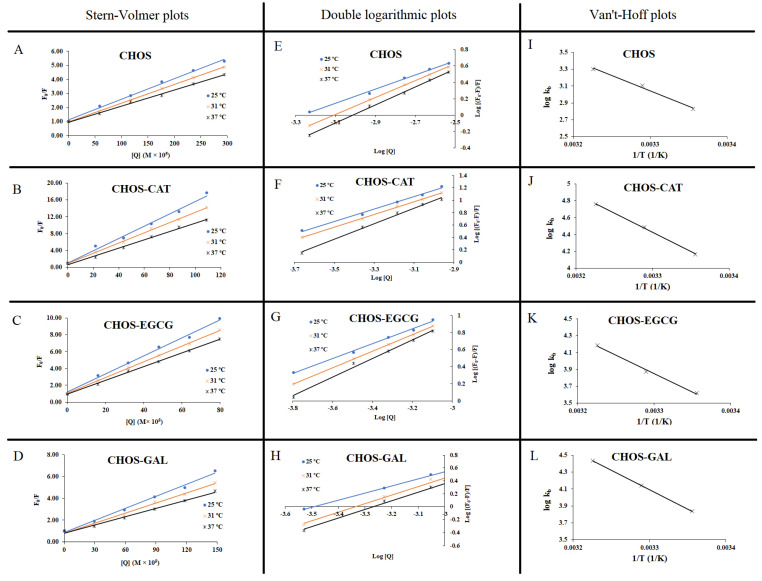
Stern-Volmer (**A**–**D**) and double logarithmic plots (**E**–**H**) for fluorescence quenching at 25 °C, 31 °C, and 37 °C, and Van ’t-Hoff plots for thermodynamic parameters (**I**–**L**) of PPO in the presence of CHOS and different CHOS-PPN conjugates at varying concentrations. CHOS: chitooligosaccharide; CHOS-CAT: chitooligosaccharide-catechin; CHOS-EGCG: chitooligosaccharide-epigallocatechin gallate; and CHOS-GAL: chitooligosaccharide-gallic acid.

At various temperatures (25, 31, and 37 °C), the fluorescence-quenching mechanism of different samples toward PPO was elucidated. Generally, there are two types of fluorescence quenching mechanisms, so called dynamic and static [21]. In dynamic quenching, increasing temperature may improve collisional quenching, but in static quenching, the higher temperature may improve complex stability. Generally, the temperature and excited lifetime govern the type of fluorescence quenching [22]. Stern–Volmer (SV) quenching constant (*K_SV_*) of samples was obtained via SV plots (Table 2 and Figure 3A–D). CHOS and its conjugates showed linearity when F_0_/F was plotted against sample concentration (Q), indicating that either dynamic or static quenching occurred toward PPO in the presence of samples. However, the selection of one quenching mechanism is determined by altered *K_SV_* value as a function of temperature. *K_SV_* of CHOS, CHOS-CAT, CHOS-EGCG, and CHOS-GAL were decreased when the temperature was increased to 37 °C (*p* < 0.05). This result followed a static quenching. Also, hydrophobic interaction and hydrogen bonding with inhibitor could be related to the initial inhibition phase. CHOS-CAT had the highest *K_SV_* followed by CHOS-EGCG, CHOS-GAL, and CHOS, respectively (Table 2). Despite the temperature change, all conjugated samples showed higher *K_SV_* values than CHOS (*p* < 0.05). The result suggested a stronger bonding between enzyme and CHOS-PPN conjugates [23]. The biomolecular quenching constant (*kq*) of PPO by CHOS or CHOS-PPN conjugates was in the order of 10^13^ L/mol/s (Table 2 and Figure 3E–H), which was higher than the dynamic quenching rate constant (2 × 10^10^ L/mol/s), regardless of the temperature used [24]. Therefore, static quenching was suggested for all samples, which was in line with *K_SV_*. 

### 2.2. Thermodynamic for Interaction Forces between PPO and CHOS or CHOS-PPN Conjugates

∆*H*°, ∆*S*°, and ∆*G*° from Van ’t Hoff equation were used to elucidate non-covalent interactions, specifically van der Waals forces, electrostatic force, hydrophobic interaction, and hydrogen bonding between enzyme and inhibitors [13]. ∆*H*° and ∆*S*° were determined from the slope and intercept of the linear scatter plot between log kb and 1/T, respectively (Figure 3I–L). CHOS-CAT exhibited positive ∆*H*° and ∆*S*° (Table 2), which was plausibly related to hydrophobic interaction with PPO due to the presence of the benzene ring of CAT [25]. Similarly, other samples had positive ∆*H*° and ∆*S*°. The results were consistent with fluorescence quenching. Furthermore, the highest ∆*H*° and ∆*S*° were noticed for CHOS-CAT, which might be due to the higher kb value and specificity toward PPO. This led to the interaction between CHOS-CAT and hydrophobic amino acid present at the active site of PPO during the later stage of inhibition. Nevertheless, some reports suggested the hydrophobic interactions during the initial stage of inhibition and subsequently by hydrogen bonding or vice versa [13]. Also, a positive Δ*H*° suggested that inhibitor binding with PPO was an endothermic process. Moreover, all samples had ∆*G*° < 0 (Table 2). Reactions with a negative ∆*G*° could occur spontaneously [13]. On the other hand, process with a positive ∆*G*° requires energy input (non-spontaneous) [26]. 

### 2.3. Molecular Docking of proPPO with CHOS and Different CHOS-PPN Conjugates

**Figure 4:**
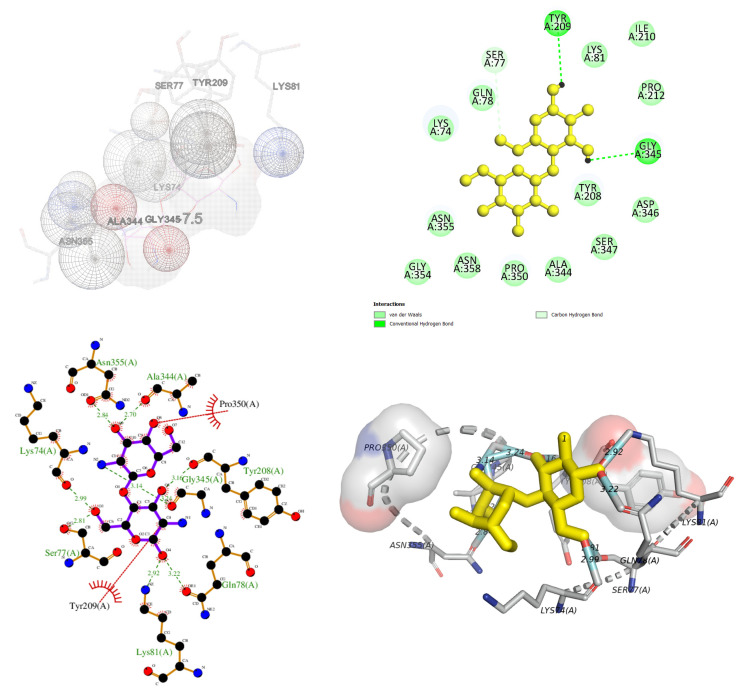
Molecular docking for interactions between chitooligosaccharide and polyphenoloxidase.

**Figure 5:**
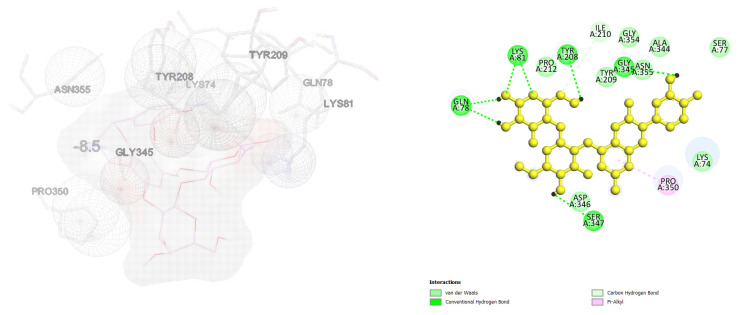

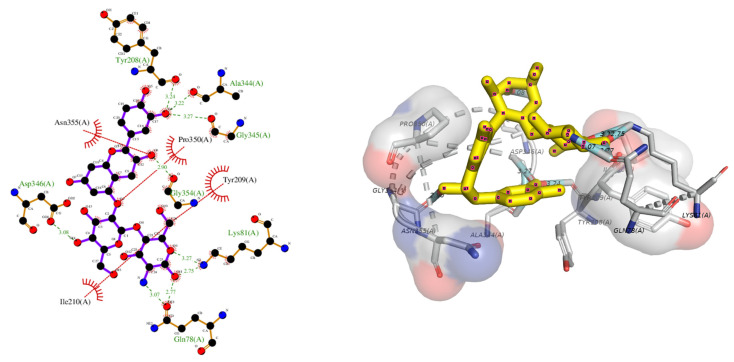
Molecular docking for interactions between chitooligosaccharide-catechin and polyphenoloxidase.

**Figure 6:**
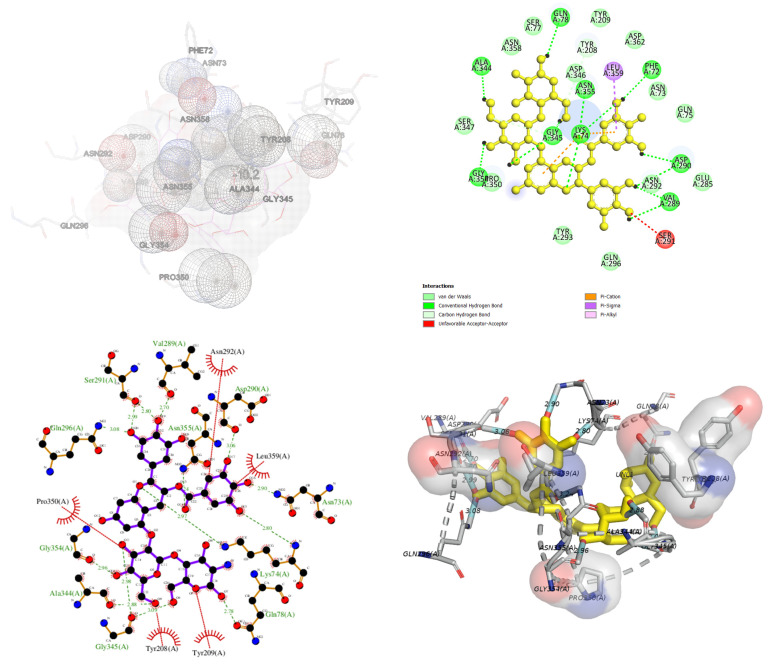
Molecular docking for interactions between chitooligosaccharide-epigallocatechin gallate and polyphenoloxidase.

**Figure 7:**
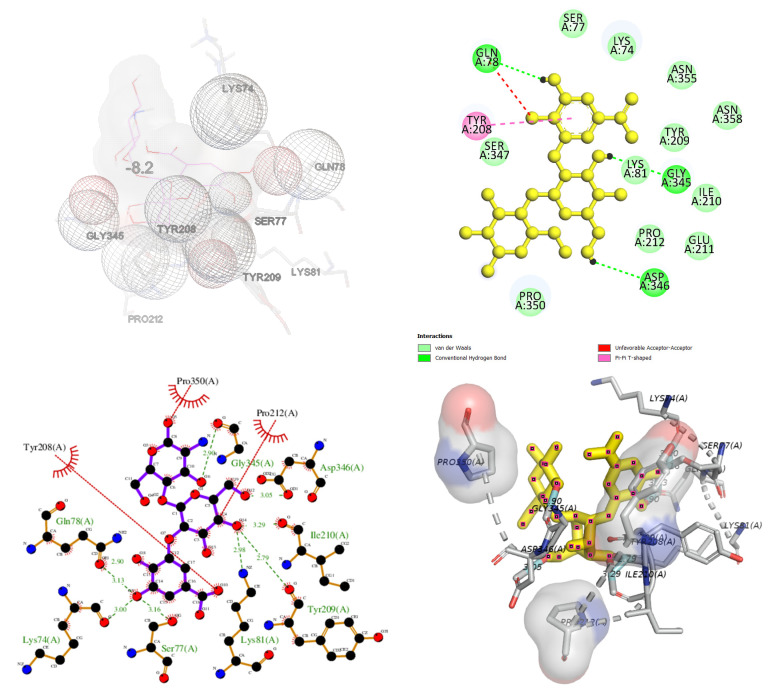
Molecular docking for interactions between chitooligosaccharide-gallic acid and polyphenoloxidase.

The inhibition mechanism of CHOS, CHOS-CAT, CHOS-EGCG, and CHOS-GAL toward proPPO was analyzed using molecular docking. In general, proPPO is the predominant form of PPO found in shrimp or other crustaceans. The most stable docked confirmations had a binding affinity of −7.5, −8.5, −10.2, and −8.2 kcal/mol for CHOS, CHOS-CAT, CHOS-EGCG, and CHOS-GAL, respectively. CHOS had the highest binding energy for proPPO, followed by CHOS-GAL, CHOS-CAT, and CHOS-EGCG, respectively. The lowest binding affinity (−10.2 kcal/mol) was noticed for CHOS-EGCG, whereas CHOS had the maximum binding affinity (−7.5 kcal/mol). This suggested the higher binding efficacy of CHOS-EGCG with proPPO, compared to others owing to the conjugation of PPN on the CHOS backbone. Figure 4, Figure 5, Figure 6 and Figure 7 depict the interaction models of various samples with binding sites on proPPO. The obtained results revealed that CHOS interacted with amino acid residues of proPPO, including Tyr209 and Gly345, via hydrogen bonding (Figure 4). Furthermore, van der Waals forces between CHOS and Tyr208 of proPPO were also noticed (Figure 4). Hydrogen bonding between CHOS-CAT and Tyr208 present in the active site of proPPO was also observed. Tyr208 and Tyr209 separated the active site cavity from the cleft. Therefore, these residues could control the activity of this enzyme [27]. Additionally, aromatic rings of CAT in CHOS-CAT displayed different π-interactions with Pro350. Also, the stability of the CHOS-CAT and proPPO complex might be enhanced by hydrogen bonding with other amino acid residues (Figure 5). CHOS-EGCG also exhibited interaction with both Tyr208 and Tyr209 residues of proPPO (Figure 6). Galloyl moiety of EGCG had π-cation and π-sigma interactions with Lys74 and Leu359 of proPPO, respectively (Figure 6). On the other hand, polar amino acids (Asp290, Gly345, Gly354, Ala344, and Gln78) of proPPO interacted with phenolic groups of CHOS-EGCG via hydrogen bonding [28]. Moreover, CHOS-GAL is bound with Tyr209 and other residues of proPPO via hydrophobic interactions. Furthermore, Van der Waals forces, as well as π-donor hydrogen bonding of CHOS-GAL with proPPO, were also noticed (Figure 7). The results coincided with mixed-type inhibition, where amino acid residues at both active and allosteric sites were involved in the interactions with inhibitors. Also, molecular docking results were consistent with fluorescence quenching.

## 3. Materials and Methods

### 3.1. Chemicals

All the analytical grade chemicals were used. L-β-(3,4-dihydroxylphenyl) alanine (L-DOPA) and Brij-35 were purchased from Sigma Aldrich (St. Louis, MO, USA). Ammonium sulfate, sodium chloride, sodium phosphate monobasic, and sodium phosphate dibasic were supplied by Loba Chemie Pvt. Ltd. (Mumbai, India). Gallic acid, catechin, and epigallocatechin gallate were obtained from Xi’an Julong Bio-Tech Co., Ltd. (Xi’an, China).

### 3.2. Extraction of PPO from the Shrimp Cephalothorax and Activity Assay

The cephalothorax was powdered by grinding in liquid N_2_. The obtained powder was kept in the zip-locked bag, stored at −40 °C, and used within 2 weeks. PPO extraction was performed as suggested by Sae-Leaw, Benjakul [14]. PPO activity was determined [15] using L-DOPA as a substrate. The absorbance of samples at 475 nm was taken using a microplate reader (FLUOstar Omega, BMG Labtech GmbH, Ortenberg, Germany) continuously for 15 min. One unit of activity was defined as the PPO causing an increase in the absorbance at 475 nm by 0.001 min^−1^.

### 3.3. Impact of CHOS and Different CHOS-PPN Conjugates on PPO Inhibition

#### 3.3.1. Preparation of CHOS and Various CHOS-PPN Conjugates

CHOS, with a degree of polymerization and average MW of 2–8 and 0.7 kDa, respectively, was produced via the redox pair hydrolysis method using the ascorbic acid and H_2_O_2_ [29]. CHOS-PPN conjugates were prepared using the selected reaction conditions via free radical grafting method [13]. CHOS solution (1%, *w*/*v*; pH: 5) was mixed with hydroxyl radical redox solution prepared using ascorbic acid (0.10 g) and 1 M H_2_O_2_ (4 mL) at 40 °C for 10 min. Then, the mixture was stirred at room temperature for 1 h on the magnetic stirrer. Thereafter, 40% catechin (CAT), 10% epigallocatechin gallate (EGCG), or 20% gallic acid (GAL) was added to the mixture separately and incubated at room temperature for 24 h in the dark. Afterward, free CAT, EGCG, and GAL were removed via dialysis with 20 volumes of distilled water for 24 h at 4 ºC. Dialysis process was repeated twice. The dialysate was subjected to lyophilization using a freeze dryer. CHOS conjugates prepared using CAT, EGCG, and GAL were named ‘CHOS-CAT’, ‘CHOS-EGCG’, and ‘CHOS-GAL’, respectively. CHOS-CAT, CHOS-EGCG, and CHOS-GAL had conjugation efficiency of 17, 30, and 36%, respectively, as assayed by Mittal and Singh [13].

#### 3.3.2. Inhibitory Effect of CHOS and Different CHOS-PPN Conjugates on PPO Activity

CHOS and its derivatives with different concentrations (0.2–1 mg/mL) (20 μL) were added with 20 μL of crude PPO extract (1145 U/mL). The mixture was allowed to stand at 25 °C for 30 min. Then, the mixture was added with 80 µL of 0.05 M sodium phosphate buffer (pH 6.0) and thereafter the reaction was initiated at 45 °C by adding L-DOPA (15 mM; 120 µL). The absorbance (475 nm) of samples was read using a microplate reader continuously for 15 min. Instead of samples, distilled water was used to prepare the control in the same manner. Percent inhibition was computed based on PPO activity of control (*A*) and residual PPO activity in the presence of sample (*B*) using the following equation:

$$Inhibition\;\left(\%\right)=\left[\frac{A-B}{A}\right]\times 100$$

IC_50_ represents the half-maximum inhibitory concentration of samples, which reduced 50% of PPO activity and was computed by nonlinear regression using GraphPad Prism 9.5 (GraphPad Software, Boston, MA, USA).

### 3.4. Study on Inhibition Kinetics and Mode of Action of CHOS and Various CHOS-PPN Conjugates toward PPO

#### 3.4.1. Inhibition Kinetics

The inhibition mode of CHOS, CHOS-CAT, CHOS-EGCG, and CHOS-GAL at different concentrations (0.2–1 mg/mL) toward PPO was determined. L-DOPA at various concentrations (2.5, 5, 10, 15, 20, and 25 mM) was used as substrate. The absorbance at 475 nm was recorded every 5 min using a microplate reader to monitor the formation of dopachrome. The initial reaction rate (*v*) was calculated at various concentrations of both the sample and substrate. The inhibition kinetics were determined using three different models as follows:

Lineweaver-Burk (LB) equation:

$$\frac{1}{v}=\frac{K_{m}}{v_{max}}\left(\frac{1}{S}\right)+\frac{1}{v_{max}}$$

Dixon equation:

$$\frac{1}{v}=\frac{K_{m}}{v_{max}}\left(\frac{1}{S}\right)+\frac{\left(I\right)K_{m}}{K_{ic}\;v_{max}}$$

Cornish-Bowden (CB) equation:

$$\frac{\left(I\right)}{v}=\frac{K_{m}}{v_{max}}\left(1+\frac{\left(I\right)}{K_{ic}}\right)+\frac{\left(I\right)}{v_{max}}\left(1+\frac{\left(I\right)}{K_{iu}}\right)$$

where *v*: initial reaction rate at different concentrations of the substrate; *v_max_*: maximum reaction rate; *S*: concentration of substrate; *I*: concentration of inhibitor. *K_m_*, *K_ic_*, and *K_iu_* represent Michaelis, competitive inhibition, and uncompetitive inhibition constants, respectively.

#### 3.4.2. Intrinsic Fluorescence Spectra 

The fluorescence spectra of PPO in the presence of various samples were determined using a spectrofluorophotometer (Shimadzu RF-1501, Shimadzu Corporation, Kyoto, Japan) [30]. Firstly, samples were prepared in distilled water to obtain several levels (0.2–1 mg/mL). Then, each sample was mixed with PPO (1145 U/mL) at a 1:1 ratio (1.5 mL each) and kept for 5 min for equilibration at different temperatures (25, 31, and 37 °C). The excitation wavelength at 280 nm and emission wavelength range of 300–500 nm were used to measure fluorescence intensity. The excitation and emission bandwidths were both set at 10 nm. The appropriate blank (sodium phosphate buffer) was subtracted for background correction. 

Before analysis, all the fluorescence intensities were corrected as follows [2]:

$$F_{c}=F_{m}10^{(A_{1}+A_{2})/2}\;$$

where *F_c_* and *F_m_* are corrected and measured fluorescence. *A*_1_ and *A*_2_ are the absorbances of samples at excitation and emission wavelengths, respectively. 

Fluorescence-quenching was computed using the Stern–Volmer (*SV*) equation:

$$\frac{F_{o}}{F}=1+k_{q}\tau_{o}\left(Q\right)=1+K_{SV}\left(Q\right)$$

where *F_o_* and *F* are fluorescence intensity without and with the sample, respectively; *k_q_* and *τ_o_* are bimolecular quenching constant and the average lifetime of the fluorophore in the absence of quencher (*τ_o_* = 10^−8^ s), respectively [23]. *Q* and *K_SV_* are the concentration (M) of the sample and fluorescence-quenching constant, respectively. In addition, *K_b_* is the binding constant, and *n* is the number of binding sites. *K_b_* and *n* were determined from the following equation:

$$log\frac{F_{o}-F}{F}=\log k_{b}+n\;log\left(Q\right)$$

#### 3.4.3. Thermodynamic Parameters

Thermodynamic parameters including enthalpy change (Δ*H°*), entropy change (Δ*S°*), and free energy change (Δ*G°*) were determined using the Van ’t Hoff equation as follows:

$$\log k_{b}=-\frac{\Delta H}{2.303RT}+\frac{\Delta S}{2.303R}$$

ΔG°=ΔH°−TΔS°

where *k_b_* is the binding constant at a certain temperature and *R* is the gas constant (8.314 J/mol·K).

#### 3.4.4. Molecular Docking Studies

The docking posture between proPPO and CHOS, CHOS-CAT, CHOS-EGCG, and CHOS-GAL was simulated using AutoDock Vina version 1.1.2 and MGL Tools 1.5.7 (The Scripps Research Institute, La Jolla, CA, USA). A protein data bank (PDB) was used to retrieve the crystal structure of proPPO (PDB ID: 3WKY) from shrimp. The 3D structures of CHOS, CHOS-CAT, CHOS-EGCG, and CHOS-GAL were constructed using ACD/ChemSketch (Freeware) 2022.1.0 from ACD Labs (Advanced Chemistry Development, Inc., Toronto, ON, Canada). All ligands were geometrically optimized in Chem3D Ultra 16.0 and the energy was minimized by MM2. Before docking, protein and ligands were prepared as described by Mittal and Singh [19]. A grid box enclosed the active site with dimensions of 46 Å × 48 Å × 48 Å and a grid spacing of 0.375 Å was prepared for site-specific docking. The best conformation of proPPO and ligands showing minimum energy score were selected. The interaction between ligands and amino acid residues of proPPO was elucidated using different softwares, namely Discovery Studio (BIOVIA, Dassault Systèmes, San Diego, CA, USA), PyMol (The PyMOL Molecular Graphics System, Schrödinger LLC, New York, NY, USA), and LigPlot+v.2.1 (European Bioinformatics Institute, Wellcome Trust Genome Campus, Hinxton, UK). 

### 3.5. Statistical Analysis

All experiments were conducted in triplicate. One-way analysis of variance (ANOVA) and Duncan’s multiple range test were performed using SPSS 23 (SPSS Inc., Chicago, IL, USA) for mean comparison (*p* < 0.05).

## 4. Conclusions

Inhibition kinetic, molecular docking, and spectrofluorimetric study were performed to determine the inhibition type and binding sites of CHOS and different CHOS-PPN conjugates (CHOS-CAT, CHOS-EGCG, CHOS-GAL) toward PPO. CHOS-CAT had the highest PPO inhibitory activity and inhibited PPO via mixed-type kinetics, which included both un-competitive and competitive inhibition. CHOS-PPN conjugates and PPO interactions were mainly facilitated via hydrogen bonding, van der Waals force, and hydrophobic interaction, as elucidated by molecular docking. In addition, CHOS-PPN conjugate could interact with Tyr residues present close to the active site of proPPO and quench its intrinsic fluorescence caused by aromatic amino acid residue. Hence, melanosis could be effectively impeded via the inhibition of PPO by CHOS-PPN conjugate, especially CHOS-CAT. The use of natural additives, especially CHOS-CAT, could therefore be a safer anti-melanosis agent to alleviate blackening, especially in shrimps.

## Figures and Tables

**Table 1:** Detailed kinetics of PPO activity inhibition by CHOS and different CHOS-PPN conjugates at varying concentrations.

Samples		Concentration (mg/mL)						Inhibition Type	*K_ic_*^#^ (mg/mL)	*K_iu_*^##^ (mg/mL)
		0	0.2	0.4	0.6	0.8	1.0			
CHOS	*V*_max_ *	0.0254 ^d^	0.0253 ^c^	0.0252 ^c^	0.0240 ^b^	0.0239 ^b^	0.0228 ^a^	Mixed	0.94 ^d^	0.04 ^b^
	*K*_m_ **	7.0695 ^a^	11.0882 ^b^	11.7029 ^b^	12.1849 ^c^	14.5262 ^d^	16.1448 ^e^			
CHOS-CAT	*V*_max_	0.0254 ^e^	0.0245 ^d^	0.0235 ^c^	0.0227 ^b^	0.0216 ^a^	0.0215 ^a^	Mixed	0.58 ^b^	0.02 ^a^
	*K*_m_	7.0695 ^a^	9.6703 ^b^	10.2809 ^c^	10.6803 ^c^	12.0666 ^d^	13.1848 ^e^			
CHOS-EGCG	*V*_max_	0.0254 ^e^	0.0246 ^d^	0.0245 ^d^	0.0230 ^c^	0.0224 ^b^	0.0216 ^a^	Mixed	0.72 ^c^	0.02 ^a^
	*K*_m_	7.0695 ^a^	9.8739 ^b^	11.1338 c	11.3412 ^c^	12.2422 ^d^	13.5389 ^e^			
CHOS-GAL	*V*_max_	0.0254 ^f^	0.0251 ^e^	0.0250 ^d^	0.0239 ^c^	0.0229 ^b^	0.0221 ^a^	Mixed	0.83 ^a^	0.02 ^a^
	*K*_m_	7.0695 ^a^	10.1118 ^b^	10.9176 ^c^	11.1418 ^d^	13.1848 ^e^	14.9964 ^f^			

CHOS: chitooligosaccharide; CHOS-CAT: chitooligosaccharide-catechin; CHOS-EGCG: chitooligosaccharide-epigallocatechin gallate; and CHOS-GAL: chitooligosaccharide-gallic acid. Data are presented in mean (n = 3). Different lowercase superscripts between different concentration of sample in same row indicate significant difference (*p* < 0.05). * *V_max_* and ** *K_m_* are expressed in ΔA min^−1^ and mg/mL, respectively. *K_ic_* ^#^ and *K_iu_* ^##^ were calculated based on Dixon and Cornish-Bowden equations, respectively.

**Table 2:** Fluorescence quenching and thermodynamic parameters of the interaction between PPO and CHOS or different CHOS-PPN conjugates at varying concentrations.

Samples	Temperature (°C)	*K_SV_* (×10^5^ L/mol)	*K_q_* (×10^13^ L/mol/s)	*K_b_* (×10^3^ L/mol)	n	Δ*H°* (kJ/mol)	Δ*S°* (J/mol/K)	Δ*G°* (kJ/mol)
CHOS	25	0.0147 ^cA^	0.0147 ^cA^	0.68 ^aA^	0.87	30.23	125.06	−7.057
	31	0.0133 ^bA^	0.0133 ^bA^	1.61 ^bA^	0.97			−7.807
	37	0.0114 ^aA^	0.0114 ^aA^	2.00 ^cA^	1.09			−8.557
CHOS-CAT	25	0.1456 ^cD^	0.1456 ^cD^	14.68 ^aD^	1.01	38.12	161.95	−10.165
	31	0.1213 ^bD^	0.1213 ^bD^	21.90 ^bD^	1.33			−11.137
	37	0.0987 ^aD^	0.0987 ^aD^	57.40 ^cD^	1.33			−12.11
CHOS-EGCG	25	0.1077 ^cC^	0.1077 ^cC^	6.86 ^aC^	0.97	37.92	159.82	−9.73
	31	0.0942 ^bC^	0.0942 ^bC^	15.33 ^bC^	1.10			−10.69
	37	0.0819 ^aC^	0.0819 ^aC^	27.03 ^cC^	1.25			−11.468
CHOS-GAL	25	0.0368 ^cB^	0.0368 ^cB^	4.18 ^aB^	1.03	36.21	151.52	−8.966
	31	0.0309 ^bB^	0.0309 ^bB^	7.43 ^bB^	1.03			−9.875
	37	0.0254 ^aB^	0.0254 ^aB^	15.39 ^cB^	1.25			−10.784

For captions: See Table 1. Data are presented in mean (n = 3). Different lowercase superscripts within the same sample in the same column indicate a significant difference (*p* < 0.05). Different uppercase superscripts within the same temperature in the same column indicate a significant difference (*p* < 0.05).

## Data Availability

The data presented in this study are available on request from the corresponding author.

## Supplementary Materials

The following supporting information can be downloaded at: https://www.mdpi.com/article/10.3390/molecules28145560/s1, Figure S1: Inhibition of PPO by CHOS and different CHOS-PPN conjugates at varying concentrations (A) and their IC_50_ (B). Bars represent standard deviation (n = 3). IC_50_ of COS cannot be detected. CHOS: chitooligosaccharide; CHOS-CAT: chitooligosaccharide-catechin; CHOS-EGCG: chitooligosaccharide-epigallocatechin gallate; and CHOS-GAL: chitooligosaccharide-gallic acid.

## Sample Availability

Samples used in this study are available from authors.
