# Supplementary material for: Inhibition Mechanism of Chitooligosaccharide-Polyphenol Conjugates toward Polyphenoloxidase from Shrimp Cephalothorax
Source: Molecules. 2023 Jul 20;28(14):5560. doi: 10.3390/molecules28145560 (PMC10385636; doi:10.3390/molecules28145560)
Supplement: Supplementary file 1 [file molecules-28-05560-s001.zip › molecules-2497992-supplementary.pdf]

Inhibition mechanism of chitooligosaccharide-polyphenol conjugates toward polyphenoloxidase from shrimp cephalothorax

Submitted to Molecules

Ajay Mittal[1], Avtar Singh[1], Bin Zhang[2], Qiancheng Zhao[3], and Soottawat Benjakul[1,4]*

[1]International Center of Excellence in Seafood Science and Innovation, Faculty of Agro-Industry, Prince of Songkla University, Hat Yai, Songkhla-90110, Thailand

[2]Key Laboratory of Health Risk Factors for Seafood of Zhejiang Province, College of Food Science and Pharmacy, Zhejiang Ocean University, Zhoushan-316022, China

[3]School of Food Science and Engineering, Dalian Ocean University, Dalian-116023 China

[4]Department of Food and Nutrition, Kyung Hee University, Seoul-02447, Republic of Korea

*To whom correspondence should be addressed

Email: soottawat.b@psu.ac.th

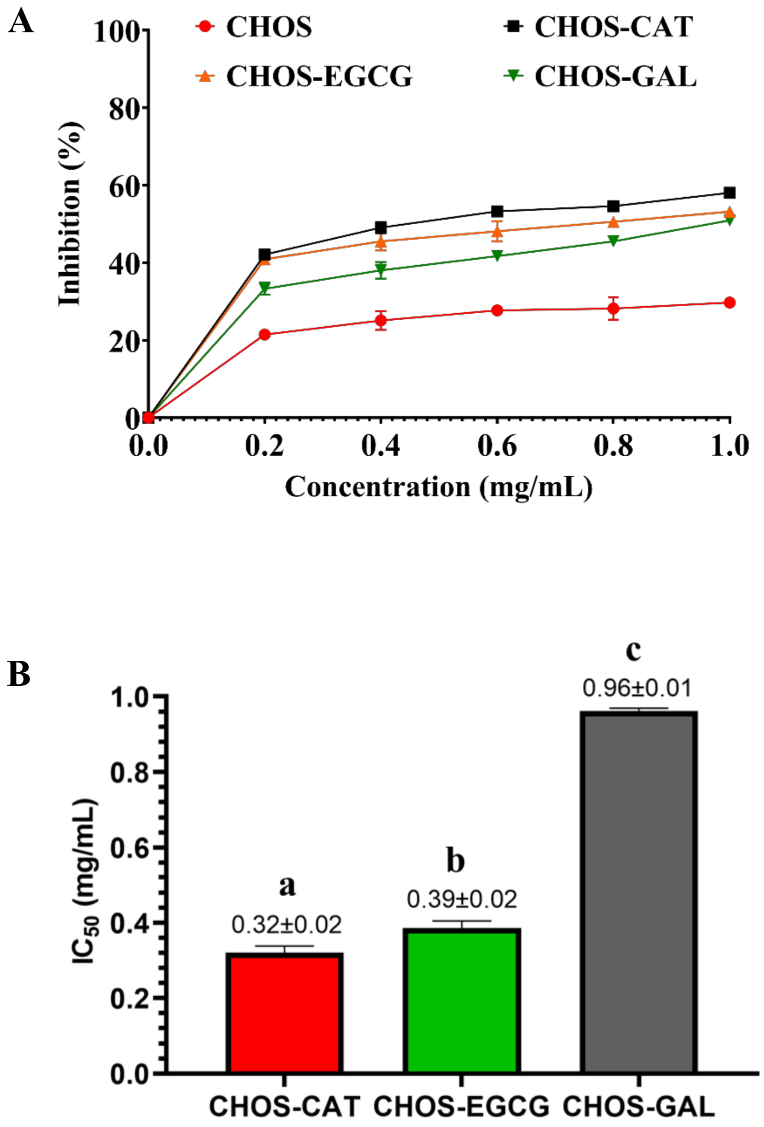

Figure S1: Inhibition of PPO by CHOS and different CHOS-PPN conjugates at varying concentrations (A) and their IC_{50} (B). Bars represent standard deviation (n=3). IC_{50} of COS cannot be detected. CHOS: chitooligosaccharide; CHOS-CAT: chitooligosaccharide-catechin; CHOS-EGCG: chitooligosaccharide-epigallocatechin gallate; and CHOS-GAL: chitooligosaccharide-gallic acid.
